# Initial surgical results of 500 Parathyroidectomies for Hyperparathyroidism related to chronic kidney disease - mineral and bone disorder

**DOI:** 10.1590/2175-8239-JBN-3924

**Published:** 2018-06-18

**Authors:** Murilo Catafesta das Neves, Lillian Andrade da Rocha, Onivaldo Cervantes, Rodrigo Oliveira Santos

**Affiliations:** 1 Universidade Federal de São Paulo. São Paulo, SP, Brasil.

**Keywords:** Parathyroidectomy, Hyperparathyroidism, Secondary, Parathyroid Diseases, Chronic Kidney Disease-Mineral and Bone Disorder, Paratireoidectomia, Hiperparatireoidismo Secundário, Doenças das Paratireoides, Distúrbio Mineral e Ósseo na Doença Renal Crônica

## Abstract

**Introduction:**

Surgical treatment of hyperparathyroidism related to chronic kidney disease is a challenging procedure even for experienced parathyroid surgeons. Over the years, adjuvant techniques have been developed to assist the medical team to improve surgical outcomes. However, medical staff in poor countries have less access to these techniques and the effectiveness of surgery in this context is unclear.

**Objective:**

verify the effectiveness of surgery for treatment of hyperparathyroidism related to chronic kidney disease without adjuvant techniques.

**Methods:**

Over a 5-years period, patients with hyperparathyroidism that had clinical therapeutic failure were evaluated for surgical treatment. Total parathyroidectomy with autograft or subtotal resection were the selected procedures. Surgeries were performed in a tertiary hospital in Brazil without the assistance of some of the adjuvant techniques that are usually applied, such as frozen section, nerve monitoring, and gamma probe. Intraoperative PTH and localization pre-operative exams were applied, but with huge restrictions.

**Results:**

A total of 518 patients with hyperparathyroidism (128 secondary and 390 tertiary) were surgically treated. Total parathyroidectomy were performed in 81.5%, subtotal in 12.4%, and 61% of patients had a surgical failure. Of all failures, only 1.4% needed a second surgery totaling 98.6% of successful initial surgical treatment. Neck hematoma and unilateral focal fold paralysis occurred in 1.9% and 1.5%, respectively.

**Conclusion:**

parathyroidectomy is a safe and reproducible surgical procedure even in the absence of adjuvant techniques.

## RESUMO

**Introdução:**

O tratamento cirúrgico do hiperparatireoidismo relacionado à doença renal crônica é um procedimento desafiador mesmo para cirurgiões de paratireoide experientes. Ao longo dos anos, técnicas adjuvantes foram desenvolvidas para ajudar a equipe clínica a aprimorar os desfechos cirúrgicos. Contudo, as equipes clínicas de países mais pobres têm menor acesso a tais técnicas, o que faz com que a eficácia da cirurgia nesses contextos não seja tão evidente.

**Objetivo:**

Verificar a eficácia da cirurgia para tratamento do hiperparatireoidismo relacionado à doença renal crônica, sem técnicas adjuvantes.

**Métodos:**

Ao longo de período de cinco anos, pacientes com hiperparatireoidismo cujo tratamento clínico não resultou em melhora foram avaliados para resolução cirúrgica. Os procedimentos selecionados foram paratireoidectomia total com enxerto autólogo ou ressecção subtotal. As cirurgias foram realizadas em um hospital terciário no Brasil sem o auxílio de algumas das técnicas adjuvantes geralmente aplicadas, como exame de congelação, monitorização neurofisiológica e sonda gama. Exames intraoperatórios de PTH e pré-operatório de localização foram realizados, mas com grandes restrições.

**Resultados:**

Um total de 518 pacientes com hiperparatireoidismo (128 secundários e 390 terciários) foram tratados cirurgicamente. Paratireoidectomia total foi realizada em 81,5% e subtotal em 12,4% dos casos; 61% dos pacientes apresentaram falha cirúrgica. De todas as falhas, apenas 1,4% necessitaram de uma segunda cirurgia, totalizando 98,6% de sucesso no tratamento cirúrgico inicial. Hematoma cervical e paralisia unilateral de prega vocal ocorreram em 1,9% e 1,5% dos pacientes, respectivamente.

**Conclusão:**

A paratireoidectomia é um procedimento cirúrgico seguro e reprodutível, mesmo na ausência de técnicas adjuvantes.

## INTRODUCTION

Hyperparathyroidism related to chronic kidney disease (CKD) is a common acquired clinical complication that usually presents with bone and muscle pain, increased incidence of bone fracture and deformity, and soft tissue and vascular calcifications. It is also associated with poor outcome and increased mortality rates.^1^^-^ 2 Patients with CKD can present a mineral and bone disorder (CKD-MBD) that plays a central role in the physiopathology of these complications and is regarded as a worldwide problem.^3^

Parathyroidectomy is the surgical treatment of choice in patients with hyperparathyroidism related to CKD-MBD; the patient's requirement for surgical treatment increases with longer CKD duration.^1^^-^ 2 Over the years, the surgery method has incorporated adjuvant techniques that are now considered important parts of the procedure. Cryopreservation, intraoperative nerve monitoring (IONM), parathyroid hormone (PTH) measurement during surgery (intraoperative PTH), and preoperative sestamibi imaging are examples of such techniques that are widely applied in the treatment of secondary hyperparathyroidism (SHPT).^4^

Patients with CKD-MBD in developing countries often have limited access to medications (particularly calcimimetics) and surgery. In 2011, the Brazilian Nephrology Society conducted a national survey that identified the severity of SHPT in Brazil5. The survey estimated that the country had about 9,800 patients requiring parathyroidectomy; however, fewer than 500 surgeries are performed yearly, imposing a 20-year delay in these patients' treatment.

This critical situation is mainly due to the small number of centers specialized in clinical and surgical treatment of patients with hyperparathyroidism and CKD-MBD. Regarding the safety of surgery, many centers use as an argument for not performing the procedure the lack of available adjuvant techniques.^6^

In this study, we report our experience with parathyroidectomy to treat patients with hyperparathyroidism related to CKD-MBD in a tertiary hospital to verify the effectiveness of the procedure performed without the assistance of adjuvant techniques.

## METHODS

After approval by the institution review board, the data of all patients who underwent surgery for hyperparathyroidism related to CKD-MBD between December 2010 and December 2016 at the *Hospital de Transplantes Euryclides de Jesus Zerbini* were retrospectively reviewed. The study included patients with SHPT undergoing dialysis, and patients with tertiary hyperparathyroidism (THPT) after renal transplantation (RTx).

As a tertiary care hospital, patients are referred to our institution exclusively to treat hyperparathyroidism. All patients included in the study were evaluated by the same group of nephrologists specialized in CKD-MBD treatment, who defined the clinical therapy for the entire cohort. Parathyroidectomy was performed only when clinical therapy had failed. After treatment, the patients returned to their initial dialysis centers.

Patients with SHPT were operated according to the guideline of the Brazilian Nephrology Society. The guideline recommends surgery for patients with serum PTH levels maintained persistently above 800 pg/mL in the following circumstances: (a) hypercalcemia and/or hyperphosphatemia refractory to medical treatment, (b) ectopic calcifications (soft and/or cardiovascular tissues) or calcific uremic arteriolopathy, and (c) progressive bone disease.^7^

The indication for surgery to treat THPT was based on the occurrence of severe hypercalcemia (ionized calcium > 1.80 mmol/dL) any time after RTx, persistent hypercalcemia for more than 12 months after RTx, kidney stone and/or nephrocalcinosis in a kidney graft or deterioration of the graft function associated with hyperparathyroidism at any time after RTx.^8^

If a patient had any of these indications and agreed to undergo surgery, parathyroidectomy was performed. The following data were collected for further analysis: gender, age, dialysis and RTx duration, laboratory tests including ionized calcium, phosphorus, creatinine, intact PTH, alkaline phosphatase and vitamin D, sestamibi imaging results, and ultrasonography findings.

All patients were operated by the same surgeon (MCN). The procedure of choice in most cases was total parathyroidectomy with presternal intramuscular parathyroid autologous transplantation, a technique that was reported in a previous study.^4^ Subtotal parathyroidectomy with removal of three glands and preservation of one entire gland could only be performed in a few selected patients. In such cases, parathyroid autologous transplantation was not performed. When fewer than four parathyroid glands were identified, cervical thymectomy and thyroidectomy were performed on the same side of the unidentified gland. Data related to the surgical findings were collected, including the number, size, and location of the parathyroid glands.

Surgical failure was defined when less than four parathyroid glands were located during surgery or when hyperparathyroidism persisted despite the removal of four parathyroid glands. Persistent disease was defined as the presence of elevated PTH and hypercalcemia up to 6 months after surgery.

Despite being a tertiary hospital, the procedures were performed in our institution with little adjuvant techniques. Pathological frozen section, IONM, or intraoperative gamma probe were not used in any surgery. Samples of intraoperative PTH were collected in most procedures, but due to the technical routine of the laboratory, the results were only available on the following day. Due to that, the intraoperative PTH results had no influence on the surgical procedure.

Parathyroid cryopreservation was routinely performed until June 2016. At that time, we followed a recommendation by the Discipline of Nephrology and discontinued parathyroid cryopreservation due to the high cost of the procedure and limited usefulness of the cryopreserved tissue.

The occurrence of postoperative complications was recorded, in particular the occurrence of neck hematoma and vocal cord paralysis. A minimum follow-up of 6 months was performed and data regarding laboratory tests during this period were collected. 

Institutional review board approval was obtained at *Plataforma Brasil* under the protocol number CAAE 30650514.4.0000.5505.

## STATISTICAL ANALYSIS

The statistical analysis was performed with Excel 2016 and complemented with the Real Statistics Resource Pack for Excel. Numerical variables are described as mean and standard deviation with Student's t test and analysis of variance (ANOVA) to verify differences between groups. Categorical variables are described as absolute number and percentage. P values < 0.05 were considered significant.

## RESULTS

During the 6-year period of this study, 518 patients with hyperparathyroidism related to CKD-MBD were surgically treated at our institution and presented sufficient data for analysis. Overall, 128 patients had SHPT and 390 had THPT. The number of males and females were 287 and 231, respectively, and the mean age of the patients at surgery was 48.4 ± 10.9 years.

The etiology of the CKD was unknown in 33.7% of the patients and was secondary to hypertension, glomerulonephritis, polycystic kidney disease, and diabetes in 21.0%, 15.5%, 9.21%, and 4.61% of them, respectively. Additional demographic features are presented in Table 1, along with the laboratory tests performed before surgery.

**Table 1:** Preoperative demographic characteristics of the patients in both groups. The data are presented as mean ± standard deviation (range)

	SHPT	THPT
Total number of patients	128	390
Duration of hemodialysis (months)	102 ± 49.6 (8 - 292)	72.3 ± 43.2 (0 - 256)
Time elapsed after RTx (months)	55 ± 47.3 (6 - 168)	44.6 ± 38.9 (1 - 221)
Mean values of preoperative laboratory tests		
PTH (pg/dL)	1650 ± 717 (372 - 4781)	342 ± 383 (71 - 3229)
Ionized calcium (mmol/dL)	1.29 ± 0.13 (0.93 - 1.61)	1.48 ± 0.13 (0.93 - 2.00)
Creatinine (mg/dL)		1.47 ± 0.59 (0.60 - 5.05)
25-hydroxyvitamin D (ng/mL)	25.1 ± 9.13 (10.5 - 41)	22.3 ± 13.3 (7 - 132.8)
Phosphorus (mg/dL)	5.8 ± 1.48 (2.1 - 12)	2.86 ± 0.93 (1.2 - 11.5)
Alkaline phosphatase (U/L)	637.4 ± 489.1 (66 - 2630)	126.3 ± 135.2 (1.2 - 1069)

Abbreviations: SHPT - secondary hyperparathyroidism; THPT - tertiary hyperparathyroidism; RTx - renal transplantation; PTH - parathyroid hormone. Reference values: PTH 15 - 68.3 pg/dL; ionized calcium 1.00 - 1.35 mmol/dL, creatinine 0.72 - 1.25 mg/dL (male) and 0.57 - 1.11 mg/dL (female); 25-hydroxyvitamin D > 30 ng/mL; phosphorus 2.3 - 4.7 mg/dL; alkaline phosphatase 40 - 150 U/L.

A total of 17 patients in the SHPT group (13.3%) had undergone RTx but had lost their renal graft function before parathyroidectomy (Table 1). All patients in the SHPT group were on hemodialysis at the time of the surgery. None of the patients was on peritoneal dialysis.

Regarding preoperative localization tests, ultrasonography and sestamibi imaging were not performed in five (3.9%) and seven (5.5%) patients, respectively, in the SHPT group and in 21 (5.4%) and 37 (9.5%) patients, respectively, in the THPT group. In patients evaluated with preoperative localization tests, the number of parathyroid glands correctly identified by each test is shown in Table 2.

**Table 2:** Number of parathyroid glands correctly identified by preoperative localization tests in each group

Number of parathyroid glands identified on ultrasound	SHPT	THPT
0	42 (34.1%)	74 (20.1%)
1	25 (20.3%)	136 (36.9%)
2	25 (20.3%)	100 (27.1%)
3	12 (9.8%)	37 (10.0%)
4	19 (15.4%)	22 (6.0%)
Number of parathyroid glands identified with sestamibi imaging		
0	13 (10.7%)	98 (27.8%)
1	32 (26.4%)	132 (37.4%)
2	49 (40.6%)	83 (23.5%)
3	16 (13.2%)	24 (6.8%)
4	11 (9.1%)	16 (4.5%)

The data are presented as absolute numbers and percentages. Abbreviations: SHPT - secondary hyperparathyroidism; THPT - tertiary hyperparathyroidism.

Out of the 512 parathyroid glands in 128 patients of the SHPT group, 504 (98.4%) were localized during surgery. As for 1,560 parathyroid glands in 390 patients in the THPT group, 1,524 (97.7%) were localized during surgery. Only 12 (2.3%) patients had a supernumerary parathyroid gland. None of the localization tests found or suggested the presence of a fifth parathyroid.

The superior parathyroids had a retroesophageal localization in 8.3% of the cases, while the inferior ones were in the thymic tongue in 8.2% of them. Ectopic mediastinal parathyroids occurred in 1.5% of all inferior glands, while intrathyroidal and undescended glands occurred in 0.8% and 0.3% of all glands, respectively.

Among all tissues removed during surgery, only five were not confirmed as parathyroids in the final pathological report and found to be four thyroid nodules and one lymph node. The type of surgery performed in each group is shown in Table 3.

**Table 3:** Type of surgery performed in each group and number of surgical failures. PTX - parathyroidectomy; SHPT - secondary hyperparathyroidism; THPT - tertiary hyperparathyroidism.

Initial surgical procedure	SHPT	THPT
Total PTX	120	302
Subtotal PTX	3	61
Failure	5	27
Days of hospital stay	7.1 (3 - 17)	3.8 (2 - 17)

Note: the number of surgical procedures is presented in absolute values, and days of hospital stay are presented in mean values and range (minimum and maximum).

Among all 32 surgical failures, in only one case all four glands were removed, with intraoperative PTH decline of 86%. In the remaining surgeries, fewer than four glands were removed. In nine (28%), the intraoperative PTH decreased more than 80%, in four (12.5%) it decreased between 70 - 79%, in 14 (43.7%) the decay was below 70%, and in five (15.6%) the intraoperative PTH was not measured. Only seven (21.8%) of the initial 32 patients required a second surgery at our institution, while 25 were still being closely followed up at the end of this study.

Among the patients who underwent total parathyroidectomy with parathyroid autologous transplantation, the mean intraoperative PTH decrease was 85.1 and 82.3% in the SHPT and THPT groups, respectively.

Neck hematoma developed in 10 (1.9%) patients, of whom five required urgent surgical drainage. The remaining five patients were managed clinically; surgical drains were not applied in any of the procedures. Eight (1.5%) patients developed unilateral vocal fold paralysis (three transient and five definitive); none developed bilateral vocal fold paralysis. One patient had both hematoma and vocal fold paralysis and required surgical drainage and tracheostomy. There were two (0.4%) deaths directly related to the surgery: one occurred during hospitalization after parathyroidectomy due to sepsis from a pulmonary origin, and the other occurred in a patient who had sudden death 1 month after parathyroidectomy.

Use of cryopreserved parathyroid tissue was possible in 39 (7.3%) patients. The mean PTH value after 6 months of follow-up in these patients was 22.6 pg/dL (1.6 - 68.0 pg/dL). Only one patient showed some improvement after regrafting during follow-up. There were two graft-dependent recurrences during follow-up; both occurred in the SHPT group and were treated with partial graft removal.

## DISCUSSION

Overall, parathyroidectomy is a safe surgical procedure. It has a typically low complication rate and is associated with clinical improvement and decreased mortality in patients with hyperparathyroidism.^1^^-^ 2 Despite a reduction in the number of surgical procedures worldwide, which has occurred mainly after the introduction of calcimimetics over the past decade,^9^^-^ 10 surgery still plays a very important role in the management of CKD-MBD. This may be especially true in poor and developing countries, where patients with CKD usually have limited access to the health care system, relying on surgery as the main treatment option.^5^^,^ 11

Our study has shown that even with limited technical resources, parathyroidectomy can be performed with high success rates. We had a 94% initial surgical success; taking into account that only seven patients required a second surgery, the overall success was 98.6%. Tominaga *et al*.,^12^ in a large series of 1,053 patients, have reported after the initial parathyroidectomy a rate of hyperparathyroidism persistence of 1.4%, in which cases the patients required a second surgery. In a more recent publication, the same authors showed a 1.85% persistence rate among more than 8,000 patients.^9^

The surgical success is directly associated with a meticulous identification of all parathyroid glands. Supernumerary glands, ectopic parathyroid, or misinterpretation of other structures as parathyroid glands (i.e., thyroid nodules or lymph nodes) are major causes of surgical failure.

The incidence of supernumerary parathyroid glands is reported to range from 2.5% to 30%.^13^^-^ 17 This broad difference is due to the routine removal of the cervical thymus, advocated by some authors, and the identification of microscopic parathyroid cell remnants, which can occur in up to 45% of the patients.^14^^,^ 17 ^-^ 18

We do not routinely perform bilateral cervical thymectomy, and for this reason, our rate of supernumerary gland was only 2.3%. Still, we do not encountered higher levels of recurrence in comparison with authors who perform thymectomy routinely, as shown in a previous publication.^19^

Ectopic localization of the parathyroid glands is a major concern in parathyroidectomy and is the main reason to perform preoperative imaging. However, it is still controversial whether preoperative imaging can improve surgical outcomes and whether it should be performed routinely prior to every surgery since bilateral neck dissection and identification of all parathyroid glands is the gold standard in the surgical treatment of hyperparathyroidism related to CKD-MBD.^20^^-^ 21

In our series, ultrasonography and sestamibi imaging were performed in several different centers all over the country, hindering any statistical analysis. This is probably the reason for having only a few localization imaging tests in which all four glands were localized. However, exams performed in specialized centers yield improved results. A recent meta-analysis including 471 patients with CKD-MBD has shown that sestamibi imaging had a 58% sensitivity in detecting hyperplastic glands,^21^ while other authors have shown that the sensitivity of ultrasonography and sestamibi imaging combination ranges from 73% to 93%.^20^

Despite the exams heterogeneity and the poor imaging quality, we were able to locate most parathyroid glands during surgery: 98.4% and 97.7% glands were located in the SHPT and THPT groups, respectively. These rates are similar to those reported by other authors.^12^

On the other hand, the number of ectopic glands was much lower than commonly reported in the literature.^9^^,^ 12 ^,^ 22 This probably reflects differences in classification. However, there is no doubt that inferior ectopic glands are usually positioned lower within the thymus, while superior ectopic glands are located posteriorly, close to or behind the esophagus.

Less common ectopic sites can also occur and usually represent a huge challenge for surgical treatment. In our series, we found 1.5% of mediastinal, 0.8% of intrathyroidal, and 0.3% of high-undescended ectopic glands. Only some of these glands had their ectopic location suggested by localization tests prior to the first surgery.

We consider that routine preoperative localization tests are not cost-effective on the first surgery to treat hyperparathyroidism related to CKD-MBD since they rarely improve the outcome. Nevertheless, these tests should always be performed after recurrence or an unsuccessful first surgical treatment.

Misidentification of parathyroid for another type of tissue is a complication that is reported rarely since few centers have an available pathologist to perform frozen section during surgery.^23^ Although our surgeries are performed in a tertiary hospital, frozen section is too time-consuming, making it difficult to perform on a regular basis. Even so, we had only five structures that were initially considered parathyroid glands but were identified as a different tissue. However, we strongly recommend the confirmation of all removed parathyroid tissue by frozen section whenever possible, especially in smaller centers.

Cryopreservation was performed routinely until June 2016. Since evidence from the literature has shown that cryopreserved cell viability decreases over time,^18^ we adopted a policy of regrafting every patient with sustained hypocalcemia 6 months or more after surgery. During this period, we performed 39 regraftings of cryopreserved parathyroid tissue (7.5% of the patients). A data analysis has shown that only one patient presented significant clinical improvement (unpublished data).

Despite legal and ethical issues, cryopreservation in our setting has faced enormous logistic and financial challenges. In addition, due to the small demand for this procedure and its limited success, we chose to interrupt cryopreservation as a routine procedure.

The role of intraoperative PTH in SHPT and THPT surgery is not as important as it is in primary hyperparathyroidism.^24^ Measurement of intraoperative PTH can indicate whether all hyperfunctional parathyroid tissue has been removed at surgery, notably when the decrease is above 80%,^25^ preventing disease persistence; it can also help identify patients with ectopic or supernumerary glands, or the removal of fewer than four glands.^18^ Ultimately, it can be helpful in an intraoperative decision to perform or not autologous transplantation. Nevertheless, intraoperative PTH measurement fails in its most important function, i.e., the long-term prediction of both hypoparathyroidism and hyperparathyroidism recurrence.^18^^,^ 24

Of all 518 surgeries performed during the study period, intraoperative PTH measurement would have prevented five (1%) out of seven reoperations, exactly the five cases in which another tissue was mistaken for a parathyroid gland. The remaining cases were due to fewer than four glands localized. Furthermore, intraoperative PTH measurement would have prevented extended neck dissection in 13 (2.5%) patients with fewer than four localized glands who presented a decrease above 70% and were still being closely followed up by the end of this study.

The complication rates in our study were comparable to those in the literature. Neck hematoma, which occurred in 1.9% of our cases, is reported to occur at a rate of 0.5% to 4.0%.^9^^,^ 12 ^,^ 26 The occurrence of permanent recurrent laryngeal nerve injury has a similar rate in the literature (around 1%).^9^^,^ 12 ^,^ 26 Only two cases (0.4%) of surgical-related mortality occurred in our study, which is within the reported rates of 0.4 to 3.0%.^9^^,^ 18

## CONCLUSION

Even with the introduction of new medications for the management of hyperparathyroidism related to CKD-MBD, surgery remains a last resource in a considerable number of patients. As shown in the present study, even in the absence of surgical adjuvant techniques, parathyroidectomy can be performed in a safe manner with high rates of surgical success and low rates of complications.
